# Influence of variable velocity slip condition and activation energy on MHD peristaltic flow of Prandtl nanofluid through a non-uniform channel

**DOI:** 10.1038/s41598-022-23308-4

**Published:** 2022-11-05

**Authors:** M. G. Ibrahim, M. Y. Abou-zeid

**Affiliations:** 1 Department of Basic and Applied Science, International Academy for Engineering and Media Science, Cairo, 11311 Egypt; 2 Department of Mathematics, Faculty of Education, Ain Shams University, Heliopolis, Cairo, 11757 Egypt

**Keywords:** Applied mathematics, Fluid dynamics

## Abstract

This study is carried out to analyze the problem of mixed convection magnet nanoflow of Prandtl fluid through a non-uniform channel with peristalsis. The external influences of activation energy and non-constant velocity slip are given full consideration. The mentioned fluid is expressed as a governing equations system, and then these equations are converted with non-dimensional parameter values to a system of ordinary differential equations. The converted system of equations is solved in terms of y and then graphs and sketches are offered using the generalized differential transform method. Graphs and results for volume friction as well as velocity profile, concentration, and temperature distributions are obtained. Results show development in the velocity profile of fluid distribution through high values of the non-constant velocity slip effect. The present study is alleged to deliver more opportunities to advance the applications of the drug-carrying system in hypoxic tumor areas with aid of identifying the flow mechanisms.

## Introduction

Lately, the important applications of non-Newtonian fluids in diverse fields have prompted investigators to study these kinds of fluids. Thus, the impact of thermal radiation on MHD Maxwell nanofluid is reported by Mahmood et al.^1^. They noticed that the rise in the temperature-dependent thermal conductivity parameter leads to elevate nanofluid temperature. Reddy and Makinde^2^ introduce a new analytical study of buoyancy forces on the influx of non-Newtonian fluids. In their investigation, they found that the volume fraction boosts with an enhancement in thermophoresis parameter $Nt$. As well, researchers, focus their concern on studying the implementations of non-Newtonian fluids in the existence of nanoparticles. Such as oil refinement implementations^3,4^, implementations of physiological systems^5^, biomedical applications^6^, drug delivery systems^7,8^, rheumatoid arthritis^9^, and digestive system^10^. Prandtl fluid is deemed an important kind of non-Newtonian fluid. It’s known as a pseudo-plastic visco-inelastic non–Newtonian fluid. Therefore, several studies included this kind of fluid. So, Akram et al.^11^ analyzed the hybrid influences of magnetic field and thermal convection on Prandtl nanofluid. In their illustration study, they observed that the velocity dwindles when $y \in \left[ {0, 0.3} \right]$ whilst, it escalates when ${\text{y}} \in \left[ {0.3,{ }1.2} \right].$ Over and above, many other analytical studies discuss the diverse applications of non-Newtonian nanofluids^12–39^.

Energy has an indispensable significant role in several implementations, like physical, engineering, and chemical areas. Thus, it has attracted the interest of investigators. In general, activation energy is the energy that must be applied to a chemical or nuclear system of latent reactants leading to a chemical reaction, or other physical phenomena. Moreover, in 1889, the term was coined by Arrhenius^40^. Shafique et al.^41^ discussed the boundary flow containing a rotating frame in the existence of activation energy. In their discussion, they observed that the activation energy is an increasing function in the fluid temperature. Gowda et al.^42^ studied a velocity distribution for the boundary layer influx in the existence of activation energy impact. Several studies include various implementations of this significant energy^43–48^.

The slip velocity is known as the difference in velocities between liquids in the vertical inflow of two-phase combinations through a pipe. Over and above, slip velocity in heart valves comes on top of its validation^49^. Nisar et al.^50^ analyzed the influences of both slip and activation energy on the peristaltic influx of Eyring Powell nanofluid. They observed that the velocity escalates with an enrichment in the slip parameter. Akbar and Nadeem^51^ propose a new model for Jeffrey’s fluid in the existence of slip impacts. They noticed in their study that the pressure rise elevates with an enhancement in the slip parameter. The slip velocity is considered in some studies because of its importance in artificial heart valves. Supplementary, see^1,6,10,26–28^. In the current study, the variable slip velocity is studied to assure its significant role in heart valves^49,50^.

Solutions to the diverse systems of equations are introduced with divergent classical techniques that are not convenient for innovation in this study. Whilst, a credible procedure utilized to solve the highly non-linear system of ordinary differential equations is named the generalized differential transform method (GDTM)^52^. This method proved to be effective in treating several kinds of equations. Also, the approximate solutions can be obtained with an error rate of up to $10^{ - 10}$ when this semi-analytical method is applied^53^, and divergent in GDTM has been appropriately contained. This current analytical study displayed a new generalization to the differential transform method to get a better solution to Prandtl nanofluid model. Several researchers studied this method, see^54,55^.

The novelty of this study is to illustrate the impacts of variable velocity slip and activation energy on MHD Prandtl nanofluid. The fluid inflows through a non-uniform channel. Distributions of velocity, temperature, concentration, and nanoparticle volume fraction are obtained by GDTM. Solutions/results are obtained without any perturbation/restrictive suggestion using GDTM. In the present paper, we construct the main results; both the nanoparticle’s volume friction ${\Omega }$ and the concentration $\varphi$ have an opposite behavior compared to the temperature behavior except that they increase or decrease with the increase of *Sc*. Physically, our model corresponds to the transport of the gastric juice in the small intestine when an endoscope is inserted through it. The formulation of the problem is introduced in "Formulation problem" section: the method of solution for the resultant system of equations is presented in "Method of solution" section. Numerical discussion and analysis of results are discussed in "Results and discussion" section. In "Conclusion" section involves the essential summarized remarks of this study.

## Formulation problem

**Figure 1:**
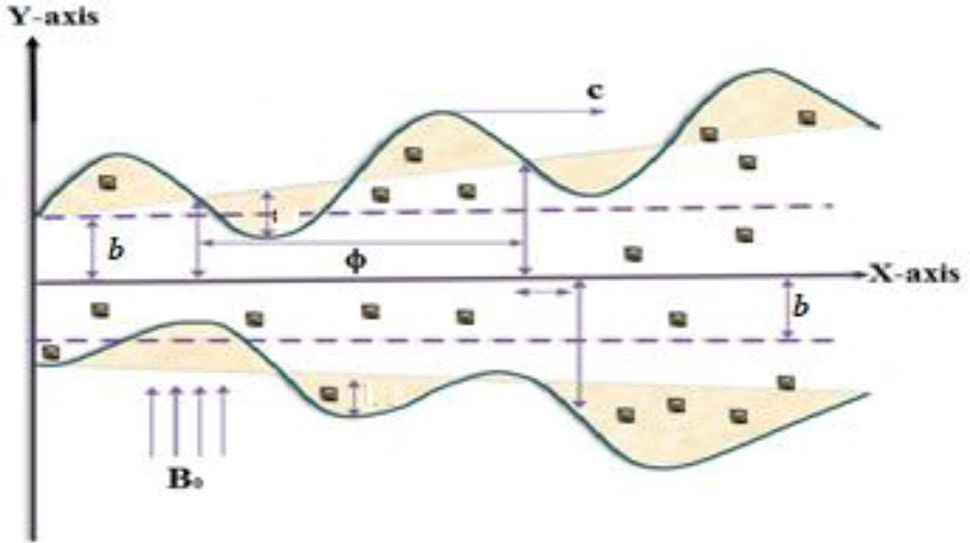
Physical flow model.

Incompressible two-dimensional MHD peristaltic flow of Prandtl nanofluid in a non-uniform channel is deliberated. The fluid velocity c in the $x - axis$ coordinates, with a width $b$. The induced magnetic field is neglected while the uniform magnetic field is applied with strength $B_{0}$ perpendicular to $x - axis$ see Fig. 1.

1 $$y = \hat{h}\left( {\hat{x},\hat{t}} \right) = \pm A_{1} \left( {\hat{x}} \right) \pm b Sin \frac{2\pi }{\lambda }\left( {\hat{x} - c\hat{t}} \right)$$

$A_{1} \left( {\hat{x}} \right) = A_{0} + \overline{m}\hat{x}$, where $A_{1} \left( {\hat{x}} \right), A_{0} , \hat{x}, b, \hat{t}, c,\lambda \;{\text{and}}\;m$ are the width of the channel, the value of half-width at the inlet, the axial space, the amplitude wave, the time, the velocity of propagating wave, the wavelength, the non-uniform parameter, respectively. Indeed, at $m = 0$ the channel wall will be a uniform channel. In two-dimensional, the axial velocity of flow is $\hat{V} = \left[ {\hat{U}\left( {\hat{X}, \hat{Y},\widehat{ t}} \right), \hat{V}\left( {\hat{X}, \hat{Y},\widehat{ t}} \right), 0} \right].$

The Prandtl fluid is offered as in^11–13^:

2 $${\varvec{S}} = \left[ {\frac{{A sin^{ - 1} \left[ {\frac{1}{B}\left( {\frac{\partial u}{{\partial y}}} \right)^{2} + \left( {\frac{\partial v}{{\partial x}}} \right)^{2} } \right]^{\frac{1}{2}} }}{{\left[ {\left( {\frac{\partial u}{{\partial y}}} \right)^{2} + \left( {\frac{\partial v}{{\partial x}}} \right)^{2} } \right]^{\frac{1}{2}} }}} \right]\frac{\partial u}{{\partial y}},$$

where $A$ and $B$ are the constants of the Prandtl fluid tensor.

Rosseland’s approximation is designated as:

3 $$q_{r} = - \frac{{ - 4\sigma^{*} }}{{3 k^{*} }}\frac{{\partial T^{4} }}{\partial y},$$

The equations of motion of incompressible flow in a two-dimensional laboratory frame $\left( {\hat{X}, \hat{Y}} \right)$ are as follows:

4 $$\frac{{\partial \hat{U}}}{{\partial \hat{X}}} + \frac{{\partial \hat{V}}}{{\partial \hat{Y}}} = 0,$$

5 $$\begin{aligned} \rho_{f} \left( {\frac{\partial }{{\partial \hat{t}}} + \hat{U}\frac{\partial }{{\partial \hat{X}}} + \hat{V}\frac{\partial }{{\partial \hat{Y}}}} \right)\hat{U} & = - \frac{{\partial \hat{P}}}{{\partial \hat{X}}} + \frac{\partial }{{\partial \hat{X}}}{\varvec{S}}_{{\hat{X}\hat{X}}} + \frac{\partial }{{\partial \hat{Y}}}{\varvec{S}}_{{\hat{X}\hat{Y}}} - \sigma B_{0}^{2} \hat{U} + g\left\{ {\left( {1 - {\hat{\Upsilon }}_{0} } \right)\rho_{f} \left\{ {\beta_{{\hat{T}}} \left( {\hat{T} - \hat{T}_{0} } \right)} \right.} \right. \\ & \quad \left. {\left. { - \left( {\rho_{p} - \rho_{f0} } \right)\left( {{\hat{\Upsilon }} - {\hat{\Upsilon }}_{0} } \right) + \beta_{{\hat{C}}} \left( {\hat{C} - \hat{C}_{0} } \right)} \right\}} \right\}, \\ \end{aligned}$$

6 $$\rho_{f} \left( {\frac{\partial }{{\partial \hat{t}}} + \hat{U}\frac{\partial }{{\partial \hat{X}}} + \hat{V}\frac{\partial }{{\partial \hat{Y}}}} \right)\hat{V} = - \frac{{\partial \hat{P}}}{{\partial \hat{Y}}} + \frac{\partial }{{\partial \hat{Y}}}{\varvec{S}}_{{\hat{Y}\hat{Y}}} + \frac{\partial }{{\partial \hat{X}}}{\varvec{S}}_{{\hat{Y}\hat{X}}} - \sigma B_{0}^{2} \hat{V},$$

7 $$\begin{aligned} \left( {\rho c} \right)_{f} \left( {\frac{\partial }{{\partial \hat{t}}} + \hat{U}\frac{\partial }{{\partial \hat{X}}} + \hat{V}\frac{\partial }{{\partial \hat{Y}}}} \right)\hat{T} & = k\left( {\frac{{\partial^{2} \hat{T}}}{{\partial \hat{X}^{2} }} + \frac{{\partial^{2} \hat{T}}}{{\partial \hat{Y}^{2} }}} \right) - \frac{{\partial \hat{q}_{r} }}{\partial y} + \left( {\rho c} \right)_{p} \left\{ {D_{B} \left( {\frac{{\partial {\hat{\Upsilon }}}}{{\partial \hat{X}}}\frac{{\partial \hat{T}}}{{\partial \hat{X}}} + \frac{{\partial {\hat{\Upsilon }}}}{{\partial \hat{X}}}\frac{{\partial \hat{T}}}{{\partial \hat{X}}}} \right)} \right. \\ & \quad \left. { + \frac{{D_{{\hat{T}}} }}{{\hat{T}_{0} }}\left[ {\left( {\frac{{\partial \hat{T}}}{{\partial \hat{X}}}} \right)^{2} + \left( {\frac{{\partial \hat{T}}}{{\partial \hat{Y}}}} \right)^{2} } \right]} \right\} + \sigma \left( {\hat{C}} \right)B_{0}^{2} \left( {U^{\prime } + V^{\prime 2} } \right) + D_{{\hat{T}\hat{C}}} \left( {\frac{{\partial^{2} \hat{C}}}{{\partial \hat{X}^{2} }} + \frac{{\partial^{2} \hat{C}}}{{\partial \hat{Y}^{2} }}} \right), \\ \end{aligned}$$

8 $$\left( {\frac{\partial }{{\partial \hat{t}}} + \hat{U}\frac{\partial }{{\partial \hat{X}}} + \hat{V}\frac{\partial }{{\partial \hat{Y}}}} \right)\hat{C} = D_{{\hat{C}\hat{T}}} \left( {\frac{{\partial^{2} \hat{T}}}{{\partial \hat{X}^{2} }} + \frac{{\partial^{2} \hat{T}}}{{\partial \hat{Y}^{2} }}} \right) + D_{s} \left( {\frac{{\partial^{2} \hat{C}}}{{\partial \hat{X}^{2} }} + \frac{{\partial^{2} \hat{C}}}{{\partial \hat{Y}^{2} }}} \right),$$

9 $$\left( {\frac{\partial }{{\partial \hat{t}}} + \hat{U}\frac{\partial }{{\partial \hat{X}}} + \hat{V}\frac{\partial }{{\partial \hat{Y}}}} \right){\hat{\Upsilon }} = + \frac{{D_{{\hat{T}}} }}{{\hat{T}_{0} }}\left( {\frac{{\partial^{2} \hat{T}}}{{\partial \hat{X}^{2} }} + \frac{{\partial^{2} \hat{T}}}{{\partial \hat{Y}^{2} }}} \right) + D_{B} \left( {\frac{{\partial^{2} {\hat{\Upsilon }}}}{{\partial \hat{X}^{2} }} + \frac{{\partial^{2} {\hat{\Upsilon }}}}{{\partial \hat{Y}^{2} }}} \right),$$

Here $f,g, \beta_{{\hat{C}}} , \beta_{{\hat{T}}} ,\rho_{f} ,\hat{\user2{S}}, \hat{T}, \hat{C},\rho_{p} , \hat{\Upsilon }, D_{B} ,\rho_{f0} , D_{{\hat{T}}} ,\left( {\rho c} \right)_{p} , D_{{\hat{C}\hat{T}}} , D_{s} , \left( {\rho c} \right)_{f} ,D_{{\hat{T}\hat{C}}} ,\; {\text{and }}\frac{d}{dt}$ are the body force, the acceleration due to gravity, the fluid volumetric solutal expansion, the fluid volumetrically thermal expansion, fluid heat capacity, the stress tensor of Prandtl fluid, the flow temperature, concentration and nanoparticle volume fraction, the base fluid density, the fluid density at $T_{0}$, the nanoparticle heat capacity, the density of the particles respectively, the Brownian diffusion, the thermophoresis diffusion, the sort diffusively, the solutal diffusively, DuFour diffusively, the material time derivative, respectively.

As we recognize, $\left( {X, Y} \right)$ describes the unsteady flow in a fixed frame, but $\left( {x, y} \right)$ refers to the steady wave frame motion. The non-dimensional relation between the wave and fixed frame is as follows:

10 $$p\left( {x,y} \right) = \hat{P}\left( {\hat{X},\hat{Y},t} \right), x = \hat{X} - ct, y = \hat{Y}, u = \hat{U} - c, v = \hat{V},$$

Levy the dimensionless parameters as follows:

$\hat{x} = \frac{x}{\lambda },\hat{y} = \frac{y}{{b_{0} }}, \hat{t} = \frac{ct}{\lambda }, \hat{v} = \frac{v}{c}, u = \frac{\partial \psi }{{\partial y}}, v = - \delta \frac{\partial \psi }{{\partial x}}, \delta = \frac{{b_{0} }}{\lambda }$ is the wave number, $\hat{p} = P\frac{{b_{0}^{2} }}{\mu c\lambda }$ is the pressure, $Re = \frac{{c\rho_{f} b_{0} }}{\mu }$ is the Reynolds number, ${\Omega } = \frac{{\hat{\Upsilon } - \hat{\Upsilon }_{0} }}{{\hat{\Upsilon }_{1} - \hat{\Upsilon }_{0} }}$ is the nanoparticle fraction, $\theta = \frac{{\hat{T} - \hat{T}_{0} }}{{\hat{T}_{1} - \hat{T}_{0} }}{ }$ is the temperature, $\varphi = \frac{{\hat{C} - \hat{C}_{0} }}{{\hat{C}_{1} - \hat{C}_{0} }}$ is the concentration, $P_{r} = \frac{{\left( {\rho c} \right)_{f} \nu }}{{\kappa_{0} }}$ is Prandtl number, $Le = \frac{\nu }{{D_{s} }}$ is the Lewis number, $S_{c} = \frac{{\left( {\hat{T}_{1} - \hat{T}_{0} } \right)D_{{\hat{C}\hat{T}}} }}{{D_{s} \left( {\hat{C}_{1} - \hat{C}_{0} } \right)}}$ is the Dufour parameters, $S_{r} = \frac{{\left( {\hat{C}_{1} - \hat{C}_{0} } \right)D_{{\hat{T}\hat{C}}} }}{{\left( {\hat{T}_{1} - \hat{T}_{0} } \right)\varsigma }}{ }$ is the Soret parameter, $M = \sqrt {\frac{\sigma }{\mu }} b_{0} B_{0}$ is the Hartmann number $G_{r} = \frac{{gb_{0}^{2} \left( {1 - \hat{\Upsilon }} \right)\rho_{f} \beta_{{\hat{T}}} \left( {\hat{T}_{1} - \hat{T}_{0} } \right)}}{{\mu_{0} c}}$ is the thermal Grashof numbers, $G_{c} = \frac{{gb_{0}^{2} \left( {1 - \hat{\Upsilon }} \right)\rho_{f} \beta_{{\hat{C}}} \left( {\hat{C}_{1} - \hat{C}_{0} } \right)}}{{\mu_{0} c}}$ is the nanoparticle Grashof numbers, $G_{F} = \frac{{gb_{0}^{2} \left( {\rho_{p} - \rho_{f} } \right)b_{0}^{2} \left( {\hat{\Upsilon }_{1} - \hat{\Upsilon }_{0} } \right)}}{{\mu_{0} c}}$ is the solutal Grashof numbers, $Ln = \frac{\nu }{{D_{B} }}{ }$ is the nanofluid Lewis number, $N_{t} = \frac{{\left( {\rho c} \right)_{p} D_{T} \left( {{\hat{\text{T}}}_{1} - {\hat{\text{T}}}_{0} } \right)}}{{\hat{T}_{0} \varsigma }}$ is the thermophoresis parameters and $N_{b} = \frac{{\left( {\rho c} \right)_{p} D_{B} \left( {\hat{\Upsilon }_{1} - \hat{\Upsilon }_{0} } \right)}}{\varsigma }$ is the Brownian motion parameter, $R = \frac{{4\sigma^{*} }}{{3k^{*} }}\frac{{T_{0}^{3} }}{{\varsigma c_{f} }}$ is the thermal radiation parameter, $B_{r} = E_{c} {\text{Eckret number}} \times P_{r}$.

After dropping pars, using the long wavelength and low Reynolds number, dimensionless parameters, Eqs. (4)–(9) in wave frame becomes:

11 $$- \frac{\partial p}{{\partial x}} + \frac{\partial }{\partial y}\left( {\frac{{\beta_{1} }}{6}\left( {\frac{{\partial^{2} \psi }}{{\partial y^{2} }}} \right)^{3} + \sigma \frac{{\partial^{2} \psi }}{{\partial y^{2} }}} \right) + G_{c} \varphi + G_{t} \theta - G_{r} {\Omega } - M^{2} Cos\left( \beta \right)^{2} \left( {\frac{\partial \psi }{{\partial y}} + 1} \right) = 0,$$

12 $$\frac{\partial p}{{\partial y}} = 0,$$

13 $$\begin{aligned} & \left( {1 + R_{d} } \right)\frac{{\partial^{2} \theta }}{{\partial y^{2} }} + P_{r} N_{b} \frac{\partial \theta }{{\partial y}}\frac{\partial \varphi }{{\partial y}} + B_{r} \left( {\beta_{1} \left( {\frac{{\partial^{2} \psi }}{{\partial y^{2} }}} \right)^{3} + \sigma \frac{{\partial^{2} \psi }}{{\partial y^{2} }}} \right)\frac{{\partial^{2} \psi }}{{\partial y^{2} }} \\ & \quad + P_{r} N_{t} \left( {\frac{\partial \theta }{{\partial y}}} \right)^{2} - M^{2} \left( {\frac{\partial \psi }{{\partial y}} + 1} \right)^{2} + S_{r} \frac{{\partial^{2} {\Omega }}}{{\partial y^{2} }} = 0, \\ \end{aligned}$$

14 $$\frac{{\partial^{2} \varphi }}{{\partial y^{2} }} - \left( {\rho \theta + 1} \right) \varphi e^{{\frac{ - E}{{\left( {\rho \theta + 1} \right)}}}} + S_{c} \frac{{\partial^{2} \theta }}{{\partial y^{2} }} = 0,$$

15 $$\frac{{N_{t} }}{{N_{b} }}\frac{{\partial^{2} \theta }}{{\partial y^{2} }} + \frac{{\partial^{2} {\Omega }}}{{\partial y^{2} }} = 0.$$

Eliminate pressure from Eqs. (13) and (14) yields

16 $$\frac{{\partial^{2} }}{{\partial y^{2} }}\left( {\sigma \frac{{\partial^{2} \psi }}{{\partial y^{2} }} + \frac{{\beta_{1} }}{6}\left( {\frac{{\partial^{2} \psi }}{{\partial y^{2} }}} \right)^{3} } \right) + G_{r} \frac{\partial \theta }{{\partial y}} + G_{c} \frac{\partial \varphi }{{\partial y}} - G_{F} \frac{{\partial {\Omega }}}{\partial y} - M^{2} Cos\left( \beta \right)^{2} \frac{\partial }{\partial y}\left[ {\left( {\frac{\partial \psi }{{\partial y}} + 1} \right)} \right] = 0,$$

Here, the tensor of the present non-Newtonian fluid (Prandtl fluid) is as follows:

17 $${\mathbf{S}}_{{{\mathbf{xy}}}} = \frac{{\beta_{1} }}{6}\left( {\frac{{\partial^{2} \psi }}{{\partial y^{2} }}} \right)^{3} + \sigma \frac{{\partial^{2} \psi }}{{\partial y^{2} }},$$

$\sigma = \frac{A}{\mu B}, {\text{and }}\beta_{1} = \frac{{\alpha_{1} c^{2} }}{{B^{2} b_{0} }}$ are the parameters of Prandtl fluid.

Therefore non-constant velocity slip is occupied to sightsee the performance of mucus and secretion of layers. The velocity restraints are clear as^4^:

18 $$U^{\prime} - U_{w}^{^{\prime}} = \xi s_{xy} ,$$

In which $U_{w}^{^{\prime}}$ portrays the wall velocity, $s_{xy}$ stress tensor mechanisms, $\xi$ non-constant velocity slip parameter. No slip constraints are taken by $\xi = 0$.

19 $$\psi = 0, \frac{{\partial^{2} \psi }}{{\partial y^{2} }} = 0, \theta = 0, \varphi = 0, \Omega = 0\;at\;y = 0$$

20 $$\psi = q, \frac{\partial \psi }{{\partial y}} + \xi \left( {\beta_{1} \left( {\frac{{\partial^{2} \psi }}{{\partial y^{2} }}} \right)^{3} + \sigma \frac{{\partial^{2} \psi }}{{\partial y^{2} }}} \right) = - 1, \theta = 1, \varphi = 1,\Omega = 1 \;at\;y = 1 + mx + \beta_{2} {\text{ Sin}}\left[ {2{\uppi }x} \right],$$

## Method of solution

The fluid model of the high non-linear differential Eqs. (12–15) nominated overhead is converted with GDTM as in^52–55^, and then the recurrence relations can gain the series solutions of velocity, temperature, concentration, and volume fraction solutions. Accordingly, results/graphs are calculated for distributions of flow velocity, temperature, concentration, and nanoparticle fraction versus different values of a physical parameter of interest:

Let,

21 $$y\left( {t,f,f^{\prime } , \ldots ,f^{{({\text{n}})}} } \right) = 0.$$

Subject to the initial equations

22 $$f^{\left( k \right)} \left( 0 \right) = d_{k} , k = 0,...,n - 1.$$

The function $f\left( t \right)$ is expressed by a finite series and can be written as:

23 $$f\left( t \right) = \mathop \sum \limits_{k = 0}^{N} F\left( k \right)\left( {t - t_{0} } \right)^{\left( k \right)} , \forall t \in D.$$

The GDTM series solution for a system ((12–15)) can be obtained as,

24 $$\psi \left( y \right) = \mathop \sum \limits_{n = 0}^{N} \Psi \left[ k \right]y^{n}$$

25 $$\theta \left( y \right) = \mathop \sum \limits_{n = 0}^{N} \left[ k \right]y^{n}$$

26 $$\varphi \left( y \right) = \mathop \sum \limits_{n = 0}^{N} \Phi \left[ k \right]y^{n}$$

27 $${\Omega }\left( y \right) = \mathop \sum \limits_{n = 0}^{N} {\Upsilon }\left[ k \right]y^{n}$$

Now, the skin friction coefficient *τ*_*ω*_, the heat transfer coefficient (Nusselt number) *Nu* and the mass transfer coefficient (Sherwood number) *Sh* at the wavy wall of the outer tube, are defined, respectively, by

28 $$\tau_{\omega } = \left. {\left( {\frac{{\beta_{1} }}{6}\left( {\frac{{\partial^{2} \psi }}{{\partial y^{2} }}} \right)^{3} + \sigma \frac{{\partial^{2} \psi }}{{\partial y^{2} }}} \right)} \right|_{y = h} ,\;\;Nu = \left. {\frac{\partial \theta }{{\partial y}}} \right|_{y = h} ,\;\;Sh = \left. {\frac{\partial \varphi }{{\partial y}}} \right|_{y = h}$$

The expressions for *τ*_*ω*_, *Nu* and *Sh* have been obtained by substituting from Eqs. (24)–(26) into Eq. (28) respectively, and they have been evaluated numerically for several values of the parameters of the problem, using the software Mathematica package. The obtained results will be discussed in the next section.

## Results and discussion

In this section, the computational results are evaluated for this problem by using the Wolfram MATHEMATICA package ver. 13.1.1. The next values of humans small intestine parameters are utilized^30^

$$m = 0.5, \beta_{2} = 0.1\;{\text{cm}}/{\text{min}}, \lambda = 8.1\;{\text{cm}}.$$

**Figure 2:**
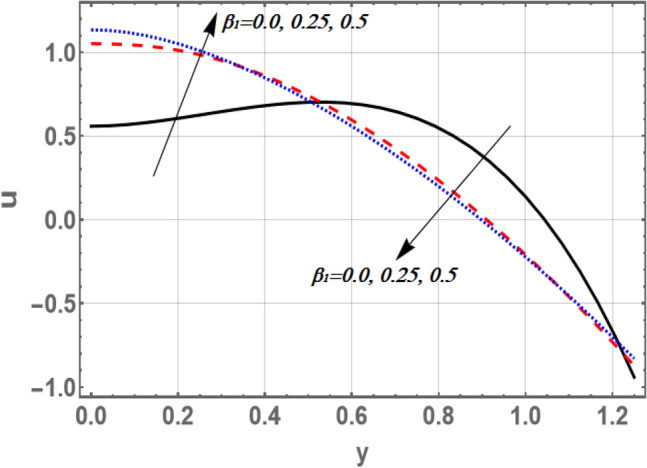
Graph of axial velocity u for different values of $\beta_{1}$.

**Figure 3:**
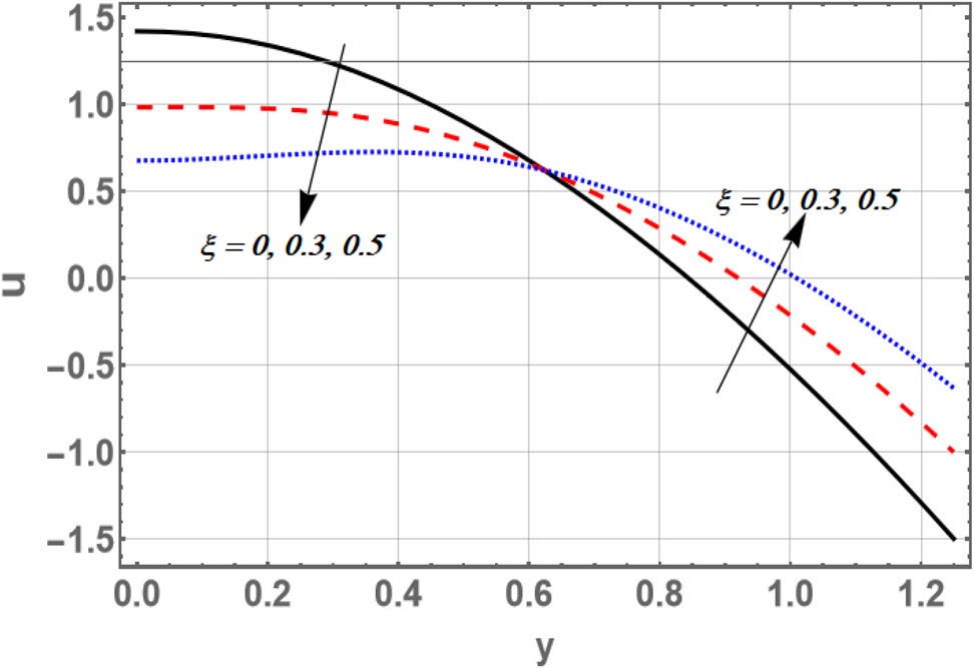
Graph of axial velocity u for different values of $\xi$.

**Figure 4:**
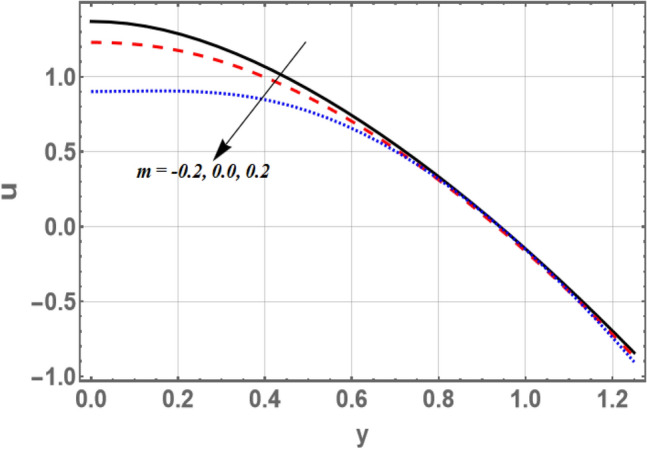
Graph of axial velocity u for different values of $m$.

Based on Eq. (27), Figs. 2 and 3 elucidate the non-material parameter $\beta_{1}$ influence and the parameter of non-constant velocity slip $\xi$ on the axial velocity *u*, respectively. It is appreciated from Figs. 2 and 3, that the axial velocity upturns as $\beta_{1}$ rises, while it declines as $\xi$ growths in the interval $y \in { }$[0, 0.6]. otherwise, it rises by snowballing $\xi$ and declines as $\beta_{1}$ grows. So, the performance of *u* in the interval $\eta$ ∈ [0, 0.6], is in contradictory manner of its behavior in the interval $\eta$ ∈ [0.6, 1.2]. It is also noted that the axial velocity for small values of $\beta_{1}$ and large values of $\xi$ increases by increasing *y* to a maximum value (to a critical point of ${\text{y}}:{\text{y }} = {\text{y}}_{0}$) subsequently, it declines. The effects of $\beta$ and $\sigma$ on *u* are found to be similar to the effects of $\beta_{1}$ in Fig. 2. Moreover, Fig. 3 depicts that the non-constant slip parameter has a dual role in phenomena on the velocity distribution. As per the newton’s law of viscosity, velocity distribution is considered a cumulative function in shear stress. The impact of divergent-convergent parameter *m* on the velocity profile *u* as a dimensionless coordinate function of $y$ is shown in Fig. 4. It is found that the axial velocity declines by aggregate values of *m*. Also, the result in Fig. 3 agrees with those obtained by^29^.

**Figure 5:**
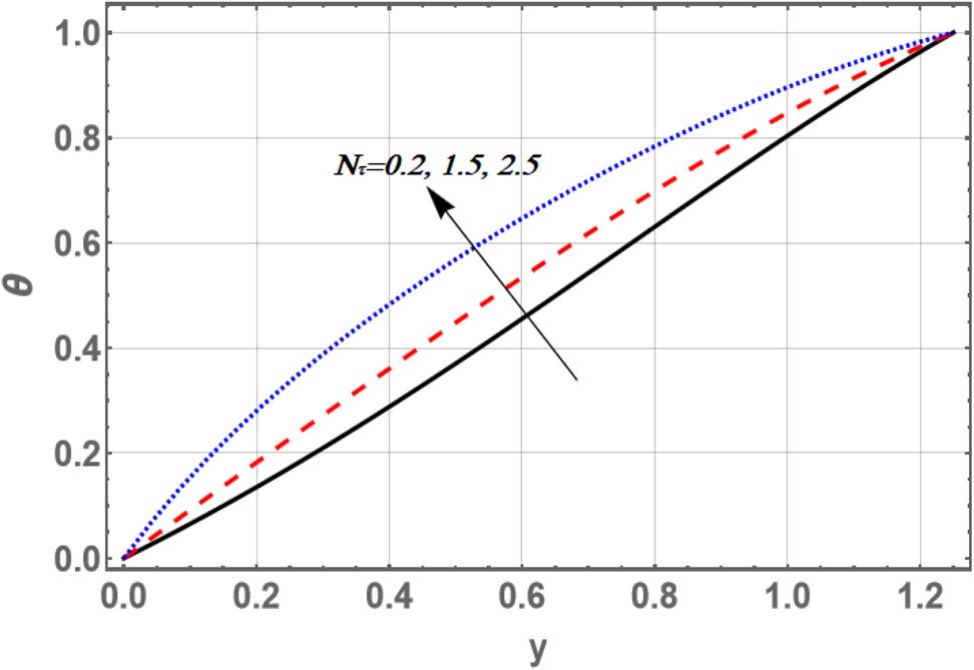
Graph of temperature $\theta$ for different values of $Nt$.

**Figure 6:**
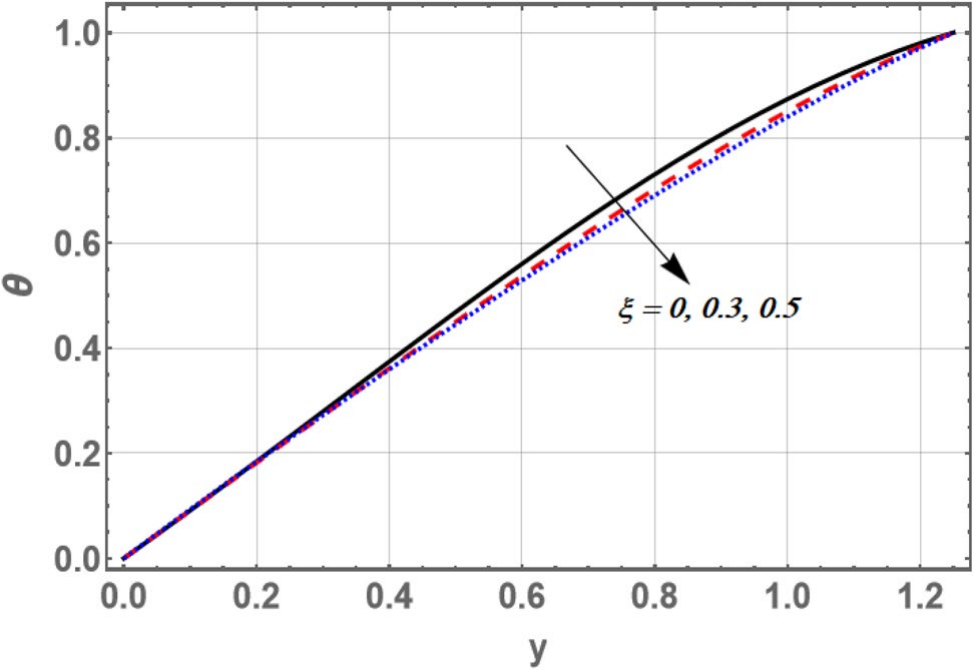
Graph of temperature $\theta$ for different values of $\xi$.

Figures 5 and 6 offer the influences of the thermophoresis parameter Nt and the non-constant slip velocity parameter $\xi$ on the temperature profile against the transverse coordinate y, respectively. It is observed that the temperature increases by increasing Nt, whereas it decreases by increasing values of $\xi$. Also, the distribution of temperature the profile is continue optimistic and negative for little values of ${\text{Nt}}$ and great values of $\xi$, there is a semi-linear relation between the axial velocity and the dimensionless coordinate y. The result in Fig. 5 shows that the improving values of thermophoresis get the nanoparticle-enhanced temperature. Both the sinusoidal layer thickness and nanoparticles concentration boundary thickness are enhanced through this result.

**Figure 7:**
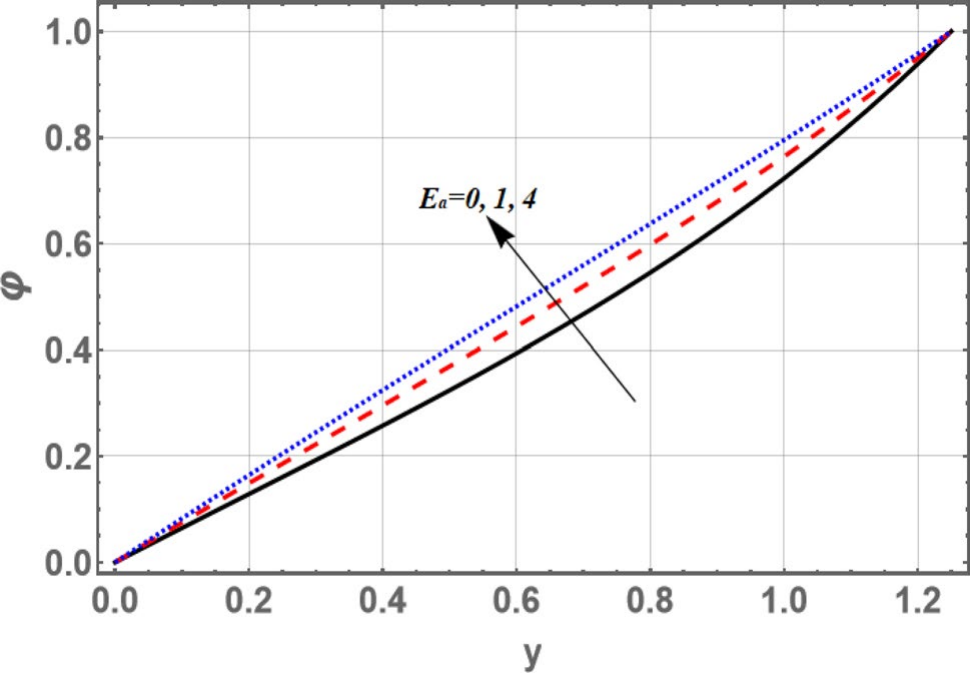
Graph of concentration $\varphi$ for different values of $E_{a}$.

**Figure 8:**
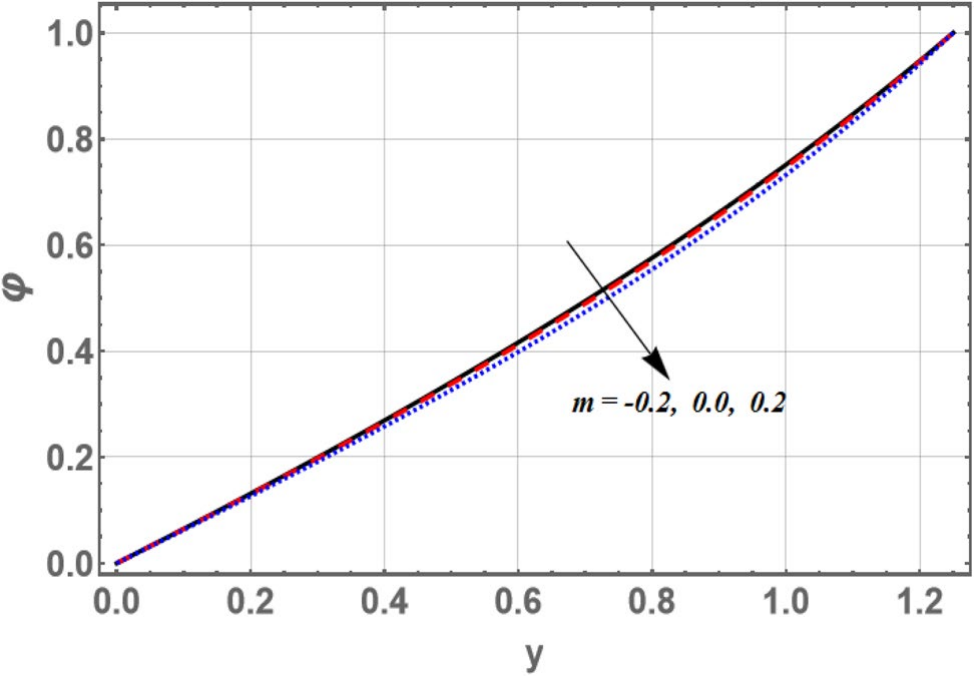
Graph of concentration $\varphi$ for different values of $m$.

**Figure 9:**
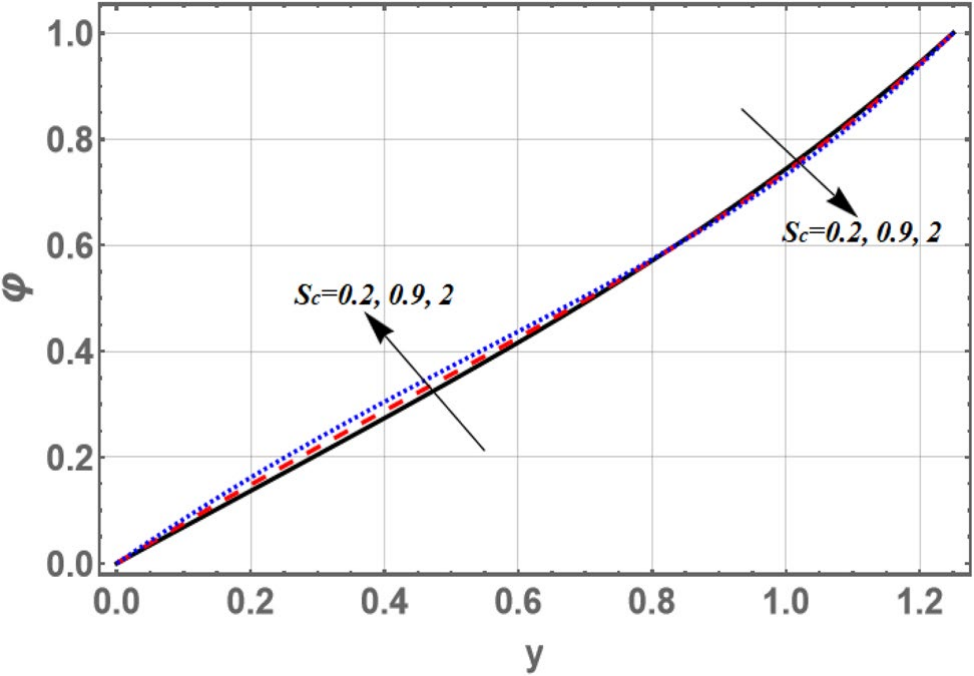
Graph of concentration $\varphi$ for different values of *Sc.*

The lowest amount of energy that allows a chemical reaction to occur is mentioned by activation energy. The effects of both activation energy parameter $E_{a}$ and non-uniform parameter *m* on the concentration distribution $\varphi$ as a function of the radial coordinate *y* is shown in Figs. 7 and 8, respectively. It is found that the concentration distribution increases by increasing $E_{a}$ and it decreases as *m* increases. Figure 9 illustrates the behavior of the concentration distribution $\varphi$ with the radial coordinate *y* for various values of Schmidt number *Sc*. It is obvious that in the interval *y* $\in$[0, 0.84]; the concentration distribution increases by increasing Sc, otherwise it decreases by increasing *Sc*. So, the performance of *f* in the interval *y* $\in$[0, 0.84], is contradictory manner of its performance in the interval *y* $\in$[0.84, 1.2] except that the curves are night boor hood to respectively other in the second interval, called, straight the channel boundary, then those acquired in the first interval. The influences of $G_{F}$ and $\sigma$ on the concentration are found to be parallel to the influence of *m* given in Fig. 8, but figures are excluded here to save space.

**Figure 10:**
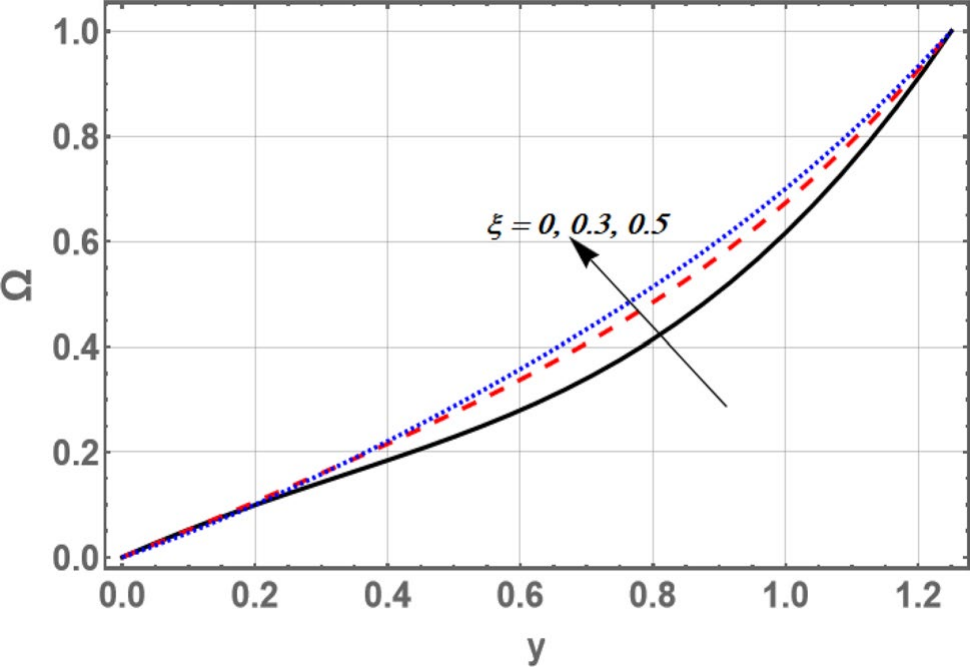
Graph of nanoparticles volume friction ${\Omega }$ for different values of $\xi$.

**Figure 11:**
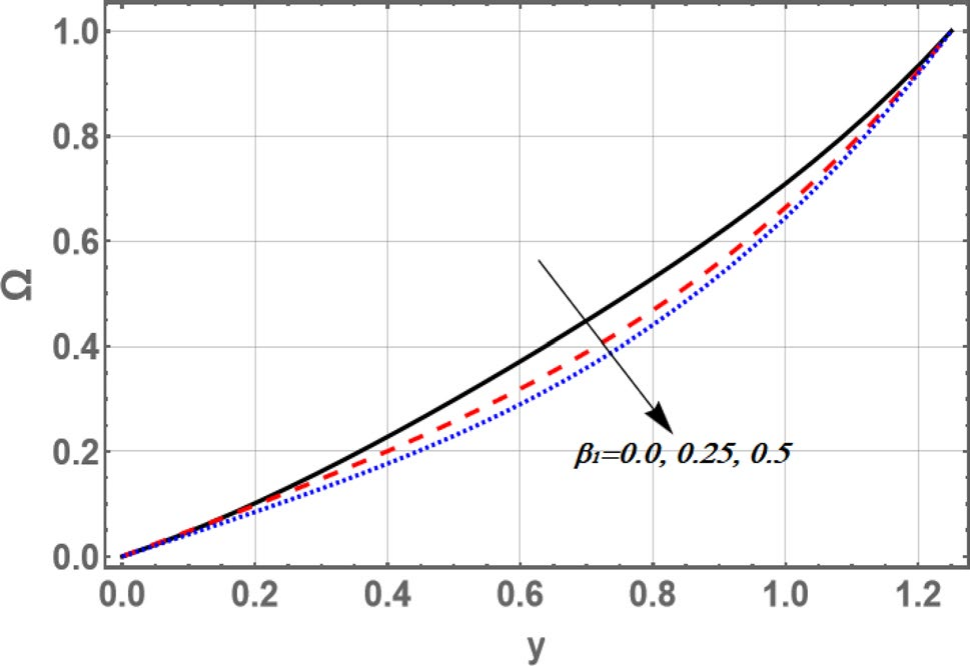
Graph of nanoparticles volume friction ${\Omega }$ for different values of $\beta_{1}$.

The variance of the nanoparticles volume friction ${\Omega }$ versus *y* for distinct values of and the variable velocity slip parameter $\xi$ and the non-material parameter $\beta_{1}$ is portrayed in Figs. 10 and 11, respectively. It is observed that the volume fraction of nanoparticles amplify with the growth in the value of $\xi$, while it decreases as $\beta_{1}$ increases.

**Figure 12:**
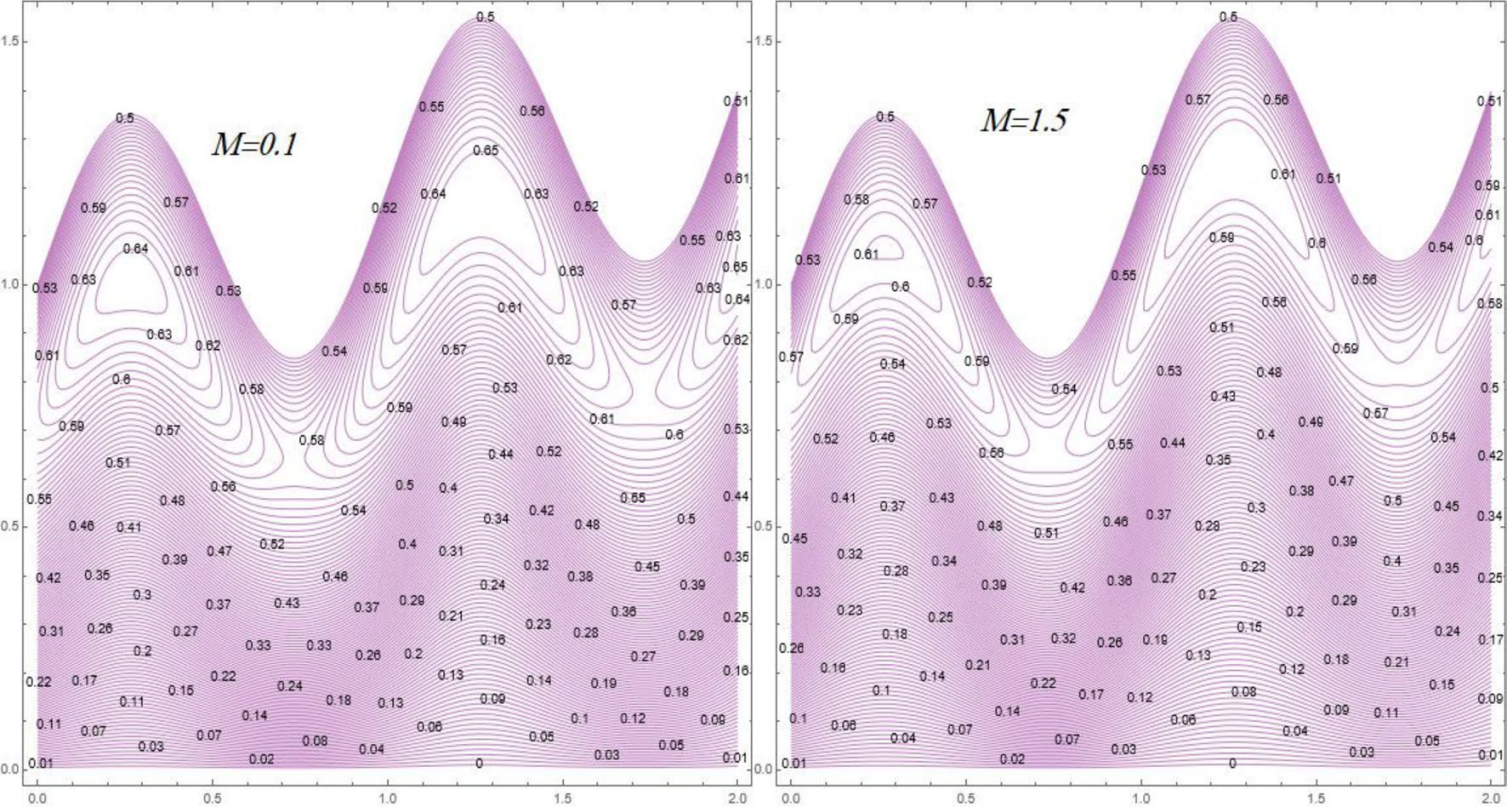
Streamlines behavior against different values of Hartmann number $\left( M \right)$.

**Figure 13:**
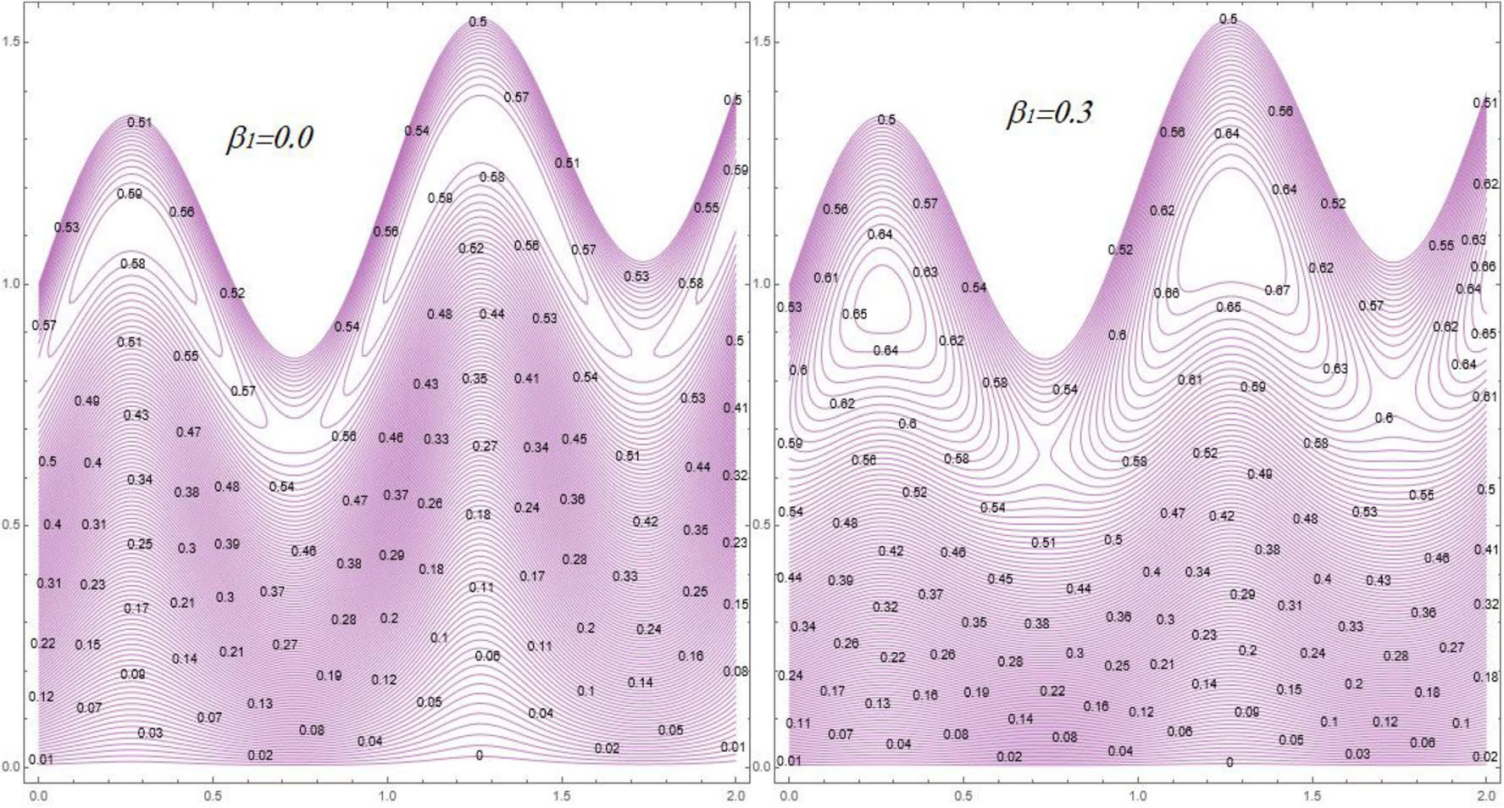
Streamlines behavior against different values of Prandtl fluid parameter $\left( {\beta_{1} } \right)$.

Contour lines distribution are graphed versus different numbers of ($M$), and ($\beta_{1}$) by Figs. 12 and 13. Figure 12 elucidates that the number of bolus/trapped zones growths with at high numbers of $M$, It’s portrayed from Fig. 13 that the behavior of the circulating bolus shrinkages under the result of high numbers of $\beta_{1}$. *Physically*, the fluid particles become more free in boluses through ejaculation, the number of trapped bolus growth bases the velocity of fluid upturns.

Table 1 presents numerical results for the skin friction coefficient $\tau_{\omega }$, Nusselt number *Nu* and Sherwood number *Sh* for various values of Prandtl number Pr and the parameters of Prandtl fluid $\beta_{1}$. It is clear from Table 1 that an increase in Pr increases the values of both *τ*_*xy*_ and Nu, while Sh decreases. Furthermore, an increase in $\beta_{1}$ gives an opposite behavior to Pr. Moreover, the result in Table 1 are in agreement with those obtained by^46^.

**Table 1:** Values of $\tau_{\omega }$, *Nu* and *Sh* for various values of $Pr$ and $\beta_{1}$.

$Pr$	$\beta_{1}$	$\tau_{\omega }$	$Nu$	$Sh$
0.2	0.1	0.01648194	0.60245875	0.71687739
0.4	0.1	0.02414264	0.62437264	0.71073276
0.6	0.1	0.03183231	0.64674878	0.70445326
0.8	0.1	0.03954870	0.66959093	0.69803765
1.0	0.1	0.04728954	0.69290279	0.69148469
2.0	0.1	0.08628082	0.81663030	0.65661820
5.0	0.1	0.20378439	1.26268139	0.529901510
0.1	0.0	0.41842978	0.71894782	0.683548140
0.1	0.1	0.04728954	0.69990279	0.691484696
0.1	0.3	− 0.44366200	0.69741969	0.692416354
0.1	0.5	− 0.87226902	0.69491969	0.699777176
0.1	0.7	− 1.25184782	0.69001969	0.705913064
0.1	0.9	− 1.59019230	0.68981969	0.710234493

## Conclusion

In the present study, a non-constant slip velocity effect on magneto Nano peristaltic flow of Prandtl fluid with heat and mass transfer in a non-uniform channel with sinusoidal deformation is treated semi-numerically. External influences like radiation, Ohmic heat, and viscous dissipation are considered. The fluid equations converted with non-dimensional values to a system of ordinary system of differential equations ODEs. The converted system of equations is solved in terms of $y$ and then graphs/sketches are offered using GDTM. The present analysis can serve as a model which may help in understanding the mechanics of physiological flows^20–39^. The numerical results indicate the following:

1. The axial velocity *u* rises or declines with the growth each of $\beta_{1}$, $\xi$*, Nt,* and *Da*, whereas it declines as *m* growths.
2. The axial velocity *w* for small values of $\beta_{1}$, $\xi$*, Nt*, becomes larger with growing the radial coordinate *y* and reaches the maximum value (at a finite value of *y*: *y* = *y*_0_) after which it declines.
3. The temperature $\theta$ advances in high values of $\beta_{1}$, *Nt*, and $\sigma$ parameters however it attenuations as both $\xi$ and *m* grow.
4. Solutions/results are obtained without any perturbation/restrictive suggestion using GDTM.
5. Both the nanoparticle’s volume friction ${\Omega }$ and the concentration $\varphi$ have an opposite behavior compared to the temperature behavior except that they increase or decrease with the increase of *Sc*.
6. In future work, the influences of emerging parameters on the pressure drop across the channel will be improved in the next paper.

## Data Availability

The datasets generated and/or analyzed during the current study are not publicly available due to [All the required data are only with the corresponding author] but are available from the corresponding author on reasonable request.
